# Single-trial ERP Quantification Using Neural Networks

**DOI:** 10.1007/s10548-023-00991-8

**Published:** 2023-08-08

**Authors:** Emma Depuydt, Yana Criel, Miet De Letter, Pieter van Mierlo

**Affiliations:** 1 Department of Electronics and Information Systems, Medical Image and Signal Processing Group, Ghent University, Ghent, Belgium; 2 Department of Rehabilitation Sciences, BrainComm Research Group, Ghent University, Ghent, Belgium

**Keywords:** Event-related potentials, Latency variability, P300, N400, Single trial analysis, Neural networks

## Abstract

Traditional approaches to quantify components in event-related potentials (ERPs) are based on averaging EEG responses. However, this method ignores the trial-to-trial variability in the component’s latency, resulting in a smeared version of the component and underestimates of its amplitude. Different techniques to quantify ERP components in single trials have therefore been described in literature. In this study, two approaches based on neural networks are proposed and their performance was compared with other techniques using simulated data and two experimental datasets. On the simulated dataset, the neural networks outperformed other techniques for most signal-to-noise ratios and resulted in better estimates of the topography and shape of the ERP component. In the first experimental dataset, the highest correlation values between the estimated latencies of the P300 component and the reaction times were obtained using the neural networks. In the second dataset, the single-trial latency estimation techniques showed an amplitude reduction of the N400 effect with age and ascertained this effect could not be attributed to differences in latency variability. These results illustrate the applicability and the added value of neural networks for the quantification of ERP components in individual trials. A limitation, however, is that simulated data is needed to train the neural networks, which can be difficult when the ERP components to be found are not known a priori. Nevertheless, the neural networks-based approaches offer more information on the variability of the timing of the component and result in better estimates of the shape and topography of ERP components.

## Introduction

Event-related potentials (ERPs) are responses in the brain that result directly from a specific sensory, cognitive, or motor event and that consist of multiple peaks and troughs which are referred to as the ERP components (Luck 2014). These responses can be measured using electroencephalography (EEG), a technique in which electrical brain activity is recorded by electrodes placed on the scalp. As EEG has a high temporal resolution, ERPs are extensively used in neuroscience to study the timing of neural responses.

When recording ERPs during tasks, the EEG signals capture not only the activity associated with the stimuli but also the ongoing spontaneous brain activity and noise. Unfortunately, the amplitude of the ERP components is often small compared to the background EEG, making it challenging to extract reliable and meaningful information from the data. Signal processing techniques that improve the signal-to-noise ratio (SNR) are required to characterize ERPs accurately. One of the most commonly used approaches is averaging the EEG signals across multiple trials. This technique is based on the assumptions that event-induced responses are consistent across different trials, and that spontaneous brain activity unrelated to the event is random and can thus be attenuated by averaging. The ERP component of interest is then typically quantified by measuring the amplitude and latency of this component in the averaged ERP. Different measures can be used for this quantification, such as the amplitude and the latency of the peak voltage, or the mean amplitude and fractional area latency (Luck 2014; Hansen and Hillyard 1980; Kiesel et al. 2008). Also other analysis techniques, for instance ERP topographic analyses of variance and microstate analyses (Murray et al. 2008), have been proven effective, but still rely on single-trial averaging in most cases.

The assumption that the ERP component is identical across trials often proves invalid in practice, since both the latency and the amplitude of different ERP components show significant variability across single trials (Handy 2005; Brazier 1964). This is especially the case for later components that express more complex cognitive processing in the brain, such as the P300, N400 and P600 (Polich 2012). This variability poses difficulties for the averaging approach (D’Avanzo et al. 2011). First, to obtain robust estimates of the latency and the amplitude of the ERP component, a large number of trials needs to be included (Clayson et al. 2013). This however increases the recording time, which may be challenging for certain patient groups. Secondly, the variability in latency will smear out the component in the averaged response, possibly reducing the amplitude of the component. Because of this effect, the averaging approach is unable to provide detailed information on the mechanisms underlying differences in ERP components between subject groups. An example often used in literature is that of schizophrenic patients, who show smaller P300 amplitudes compared to healthy controls (Jeon and Polich 2003). However, as schizophrenic patients also show higher variability in reaction times (Ford et al. 1994; Roth et al. 2007), the amplitude variation in the averaged ERP waveform could in part be caused by the variability in latency jitter (Ouyang et al. 2016). This variability in latency jitter has also been observed in other populations, such as aging populations (MacDonald et al. 2008) or in individuals suffering from brain damage (Fjell et al. 2011). Variations in the amplitude of an average ERP component may be due to changes in the amplitudes of individual trials, variability in latency, or a combination of both factors (Walhovd et al. 2008). Correcting for this latency variability may help in better understanding the neural mechanisms underlying different tasks. For example, Murray et al. (2019) have shown the parietal retrieval success effect to be both variable and thresholded in older adults by compensating for the trial-to-trial latency jitter.

Many different single-trial estimation algorithms have been proposed in literature. One of the currently most widely used techniques to quantify the single-trial latency consists of an iterative approach based on template matching and was proposed by Woody (1967). The component’s latency is estimated using the cross-correlation between a template and the single trial, after which all single trials are realigned to the estimated latencies and averaged, resulting in a new template. The assumption behind this approach is that while the latency of the ERP component varies in different trials, its shape does not. This iterative scheme results in a subject-specific estimate of the shape of the ERP component, which has, however, proven to be sensitive to noise. Errors in the latency estimation can deform the shape of the new template, enlarging the error made in subsequent iterations (Möucks et al. 1988). Another drawback of this method is that it relies on the analysis of the EEG data in a single channel. Given that in most recording set-ups multiple electrodes are used and that different electrodes simultaneously capture the evoked response, only a fraction of the available information is thus used. Therefore, techniques that also consider the topographic information in the EEG data have been extensively explored. For example, the cross-correlation curves calculated in Woody’s method can be obtained for multiple electrodes and averaged, after which the peak lag is extracted from the averaged curve (Ouyang et al. 2017). A similar template-matching technique that has been used in literature is dynamic time warping (DTW) (Zoumpoulaki et al. 2015). This alignment algorithm matches the different components of the template to the single trial through local compressions and extensions of the signal, making it possible to estimate the time points in the single trial that best resemble the ERP component of interest. The algorithm can be extended to include topographic information by using a multi-dimensional generalization of the algorithm as proposed in (Shokoohi-Yekta et al. 2017). Furthermore, different spatiotemporal filters have been proposed, including multi-channel Wiener filters (Maki et al. 2015) and spatiotemporal linearly constrained minimum variance (LCMV) beamformers (van Vliet et al. 2016). A final group of techniques exploiting the spatiotemporal information in the EEG data are decomposition techniques, such as principal component analysis (PCA) (Dien 2010) and independent component analysis (ICA) (De Lucia et al. 2010). While PCA decomposes the signal into orthogonal components that capture the maximum amount of variance in the data, ICA decomposition is based on the idea that the recorded signals in the different electrodes are different mixtures of the signals generated by several independent sources in the brain and that one or a combination of multiple of these sources corresponds to the ERP component (Bugli and Lambert 2007).

It is interesting to notice that many of the methods for the quantification of ERP components in single trials have also been used in research focusing on Brain-Computer Interfaces (BCIs). Here, for each trial, a decision has to be made whether a certain ERP component is present in the data or not. Most recent advances in this field, however, have been made using deep learning techniques, such as convolutional neural networks (CNN) (Lawhern et al. 2018; Vařeka 2020), recurrent CNNs (Maddula et al. 2017) and convolutional long short-term memory (convLSTM) neural networks (Joshi et al. 2018). This research has shown that neural networks can learn the pattern of the ERP component from the data. Therefore, deep learning approaches might also be able to improve the quantification of ERP components in single trials.

This work aims to investigate the applicability of neural networks to the quantification of ERP components in single trials. Therefore, two existing neural networks described in literature for BCIs, namely EEGNet, a compact convolutional neural network introduced by Lawhern et al. (2018), and the convolutional LSTM neural network proposed by Joshi et al. (2018) will be adapted. These neural networks will be compared to other single-trial latency estimation techniques described in literature, such as iterative and non-iterative template matching using cross-correlation and DTW, an iterative and a non-iterative spatiotemporal LCMV beamformer and a decomposition-based approach using ICA. Furthermore, the different single-trial latency estimation techniques will be compared to the traditional averaging approach to assess the added value of single-trial ERP quantification by evaluating the topography and morphology of the obtained ERP components, using both simulated and experimental data. While the focus in this work lies on the P300 and N400 components, the proposed methods could easily be adapted for other ERP components.

## Material and Methods

In this section a short explanation is given on the different datasets used in this study, followed by more information on the different methods that are used for the ERP quantification in the data. These methods are split into three different groups, namely methods for ERP quantification in averaged trials, methods for single-trial ERP component quantification based on template matching and, finally, methods for single-trial ERP component quantification using neural networks. Then, an overview of the experimental pipeline is given. First, more details about the data simulation approach are given, after which the evaluation criteria for the ERP quantification that were used both on the simulated data and on the experimental datasets are presented.

### Experimental Data

In this study, two different experimental datasets were used. For the first dataset, an attentive oddball task was used in which two types of phonemes were presented to the subjects, while the second dataset consisted of a semantic sentence congruity task. The collection of both datasets in this study were carried out in accordance with the Declaration of Helsinki and were approved by the Ethical Committee of the Ghent University Hospital (BC-11771). All participants gave written informed consent.

#### Dataset 1: Oddball Task Eliciting a P300 Component

The normative dataset for phonological input collected by Aerts et al. (2013), consisting of 71 healthy subjects between 21 and 83 years old, was used in this work both as the experimental dataset and to generate the simulated trials. In the experiment, an attentive oddball paradigm for auditory phoneme discrimination was presented to the participants, who had to discriminate the deviant phoneme [g] from the standard phoneme [b]. In total, 150 stimuli of 250 ms were presented to the participants with an interstimulus interval of 2000 ms and a deviant/standard ratio of 1/4. Participants were asked to press a button each time a deviant stimulus was presented, allowing the measurement of the reaction times to the stimuli. The data was recorded using 20 electrodes at the following positions according to the international 10-20 system: Fp1, Fpz, Fp2, F7, F3, Fz, F4, F8, T3, C3, Cz, C4, T4, T5, P3, Pz, P4, T6, O1 and Oz, at a sampling rate of 500 Hz. Additional details about the data and the recording procedure can be found in Aerts et al. (2013).

#### Dataset 2: Semantic Sentence Congruity Task Eliciting an N400 Component

For the second experimental dataset involving the N400 component elicited using a semantic sentence congruity task (SSCT), the dataset recorded by Cocquyt et al. (2023) was used. Briefly, 120 sentences, half of which were semantically correct while the other half contained a semantic violation at the end, were visually presented to 110 individuals. After the final word of each sentence, the Dutch word ‘Druk’ (‘press’) appeared on the screen, asking participants to press the green (correct sentences) or red (incorrect sentences) button. In this experiment, the participant’s response was delayed to avoid an influence of the button press on the ERPs of experimental interest. The subjects were all between 21 and 84 years old, and were divided among three age groups, i.e. the young (20-39 years, n=40), middle-aged (40-59 years, n=40) and elderly ($\ge$ 60 years, n=30), to investigate the effect of aging. More details about the complete experiment can be found in Cocquyt et al. (2023).

#### Data Preprocessing

**Fig. 1:**
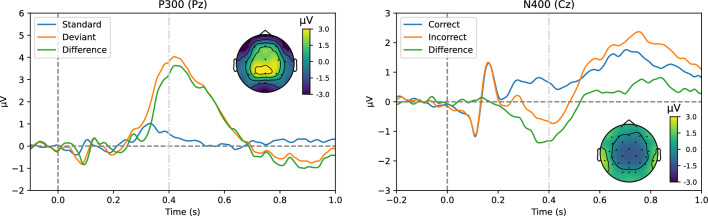
The averaged responses to the standard and deviant conditions (P300, left) and to the correct and incorrect conditions (N400, right) across all trials and subjects, as well as the difference between the conditions in the experiment for both experimental datasets. For both datasets, also the topography at 0.400 s (cf. the dotted vertical grey line) after the stimulus onset is shown

The data was preprocessed offline using the MNE-Python library (Gramfort et al. 2013). Bad electrode channels were automatically detected and removed, after which the data was band-pass filtered between 0.3 and 30 Hz (half-amplitude cut-off, 12 dB/octave roll-off), as well as notch-filtered at 50 Hz. Independent component analysis was performed to remove eye blinks and eye movements, and the data was re-referenced to an average common reference. For the oddball paradigm, the data was segmented into 1100 ms long epochs, starting from 100 ms before the stimulus onset to 1000 ms post-onset, while for the SSCT dataset, the data was segmented into epochs of 1500 ms, from 300 ms pre- to 1200 ms post-presentation of the critical words. Baseline correction was performed using the pre-stimulus window for both paradigms. Finally, automatic artefact rejection was applied, rejecting epochs where the signal exceeded $\pm 100\,\upmu \hbox {V}$, the peak-to-peak signal amplitude exceeded $150\,\upmu \hbox {V}$ or the peak-to-peak signal amplitude was less than $0.5\,\upmu \hbox {V}$. The averaged responses to the standard and deviant conditions (P300 component) and to the correct and incorrect conditions (N400 component) across all trials and all subjects are shown in Fig. 1. Also the difference between the conditions is depicted for both datasets.

### Methods for ERP Component Quantification

The methods used for the quantification of the ERP components can be split into two groups. The first group of methods follows the traditional approach where the individual trials for each subject are averaged before quantifying the latency, the topography and the shape of the ERP component using the average waveform. The second group are methods that first estimate the latency of the ERP component in single trials. Based on these estimates, the different trials are realigned for each subject before averaging, after which the obtained waveform is used to quantify the topography and shape of the ERP component.

#### Averaged Trial ERP Component Quantification

Two different techniques were used to quantify the ERP component’s latency after averaging, namely the peak latency and the 50%-area latency. For the P300 component, the signal at the Pz electrode between 250 and 650 ms post-stimulus was used, while for the N400 component the focus was on the Cz electrode using the 200–600 ms time window. More details about both methods (M1 and M2) can be found in Appendix A.1.

#### Single-trial ERP Component Quantification Based on Template Matching

Seven different approaches were selected for the single-trial latency estimation based on template matching: non-iterative and iterative template matching using cross-correlation, non-iterative and iterative template matching using DTW, a non-iterative and an iterative spatiotemporal LCMV beamformer and template matching after ICA decomposition using both a single component and multiple components. The details about how the latency of the ERP component is estimated in single trials using each of these approaches (M3 to M10) can be found in Appendix A.2.

#### Single-trial ERP Component Quantification Using Neural Networks

Finally, two deep learning approaches, namely the EEGNet network and a convLSTM neural network, were used for the quantification of the ERP components in single trials.

**M11: EEGNet, a Convolutional Neural Network** EEGnet is a compact convolutional neural network that was developed by Lawhern et al. (2018) for EEG-based BCIs. The network combines depthwise and separable convolutions, allowing the model to combine spatial and temporal information present in the data. It consists of two convolutional blocks, followed by a Softmax classification layer. The first convolutional block incorporates two sequential convolutional steps. First, a number of 2D convolutional filters are fitted to the data to capture the frequency information present in the data. A depthwise convolution is then used for the network to learn a spatial filter. This combination of convolutional layers allows the model to learn frequency-specific spatial filters for each feature map. In the next layer, batch normalization is used, before applying the exponential linear unit (ELU) non-linearity and reducing the dimensionality using Average Pooling. Finally, to reduce overfitting of the model, a dropout layer with a dropout probability of 0.5 is used. The second convolutional block consists of a separable convolution, which decouples the relationship between feature maps and reduces the number of parameters. This convolutional layer is again followed by batch normalization, ELU activation, Average Pooling and dropout, after which the features are passed to the classification block (Alvarado-Gonzalez et al. 2021).

In this work, the two convolutional blocks were similar to the original model, but the classification block was adapted to allow the estimation of the P300 latency by flattening the data and using a dense layer with linear activation instead of the Softmax classification layer. To reduce the number of parameters in the model, the EEG data was downsampled to 250 Hz. The model was fitted using the Adam optimizer (Kingma and Ba 2014), with the default parameters available in the Keras API (Chollet et al. 2015), to minimize the mean squared error loss function. Table 1 gives an overview of the final architecture of the model and the chosen parameters.

**Table 1:** Full details of the EEGNet architecture: The network starts in the first block with a temporal convolution (Conv2D) to learn the frequency filters, after which the depthwise convolutions (DepthwiseConv2D) are used to learn frequency-specific spatial filters. The secon﻿d block initially learns a temporal summary for each feature map individually (SeperableConv2D), and finally learns to mix the feature maps together. More details about the network architecture can be found in the work of Lawhern et al. (2018)

Block	Layer	No. filters	Size	No. params	Output	Activation function	Options
1	Input		1 × 20 × 101				
	Conv2D	8	1 × 64	512	(8, 20, 101)	Linear	padding = same, use_bias = False
	BatchNorm			32	(8, 20, 101)		
	DepthwiseConv2D		20 × 1	320	(16, 1, 101)	Linear	use_bias = False, number of depthwise convolution output channels = 2, max norm constraint function = 1
	BatchNorm			64	(16, 1, 101)		
	Activation				(16, 1, 101)	ELU	
	AveragePool2D		1 × 4		(16, 1, 25)		
	Dropout				(16, 1, 25)		p = 0.5
2	SeperableConv2D	16	1 × 16	512	(16, 1, 25)	Linear	padding = same, use_bias = False
	BatchNorm			64	(16, 1, 25)		
	Activation				(16, 1, 25)	ELU	
	AveragePool2D		1 × 8		(16, 1, 3)		
	Dropout				(16, 1, 3)		p = 0.5
	Flatten				(48)		
Latency	Dense	1		49	(1)	Linear	
estimation							

**M12: Convolutional LSTM Neural Network (ConvLSTM)** ConvLSTM is a specific type of recurrent neural network used for spatiotemporal predictions and was first introduced in precipitation nowcasting (Shi et al. 2015). The model can learn both spatial and temporal features at the same time. Furthermore, as convolution operations share parameters, the number of parameters in a convLSTM is reduced compared to the traditional LSTM approach. As mentioned in the introduction, the model was recently used by Joshi et al. (2018) in the area of BCIs to determine the presence of the P300 component in single trials. In this work, the proposed architecture was adapted to allow estimation of the latency of the ERP components.

**Fig. 2:**
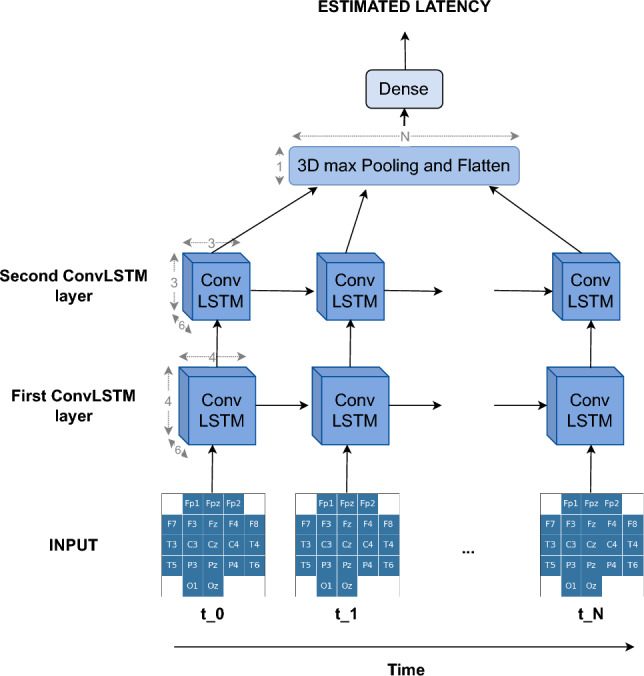
Overall visualization of the ConvLSTM network. Full details of the architecture can be found in Table 2. The network consists of two consecutive ConvLSTM layers followed by a pooling layer and a linear dense layer to estimate the latency of the ERP component. In this type of recurrent neural network, the model can hold and use information obtained from previous time points it has seen to make decisions

As convLSTM networks perform better on shorter sequences, the trials were again downsampled to 250 Hz. To preserve the spatial information present in the data, the electrodes were mapped to a 5 × 5 2D map as shown in Fig. 2. This was done for each time sample, converting each trial to a (number of time samples × 5 × 5) 3D matrix that was used as input for the neural network. The first layer of the network was a convLSTM layer in which the sequence of 2D input maps was passed through the recurrent convolutions of 6 filters with size 2 × 2. Here, the tanh-function was used as the activation function. A dropout of 0.2 was applied together with a recurrent dropout of 0.1 to reduce overfitting. In the second layer, the data was batch normalized with the batch size set to 128 samples. The convLSTM and the batch normalization layers were repeated in the third and fourth layers of this network. For each time index, the maximum value obtained across the different filters is selected, after which the data is flattened into a 1D array of size (number of time samples). The final layer of the model was a dense layer that outputs the estimate of the P300 latency. The model was again fitted using the Adam optimizer to minimize the mean squared error loss function (Kingma and Ba 2014), with the default parameters available in the Keras API (Chollet et al. 2015). A summary of the network architecture is given in Table 2 and visualized in Fig. 2.

**Table 2:** Full details of the ConvLSTM architecture. The network consists of two consecutive convolutional LSTM layers followed by a pooling layer and a linear dense layer to estimate the latency of the ERP component

Layer	No. filters	Size	No. params	Output	Activation function	Options
Input		101 × 1 × 5 × 5				
ConvLSTM2D	6	2 × 2	696	(101, 6, 4, 4)	hyperbolic tangent	dropout = 0.2, recurrent_dropout = 0.1
BatchNorm			16	(101, 6, 4, 4)		
ConvLSTM2D	6	2 × 2	1176	(101, 6, 3, 3)	hyperbolic tangent	dropout = 0.2, recurrent_dropout = 0.1
BatchNorm			12	(101, 6, 3, 3)		
MaxPooling3D		6 × 3 × 3		(101, 1, 1, 1)		
Flatten				(101)		
Dense	1		102	(1)	Linear	

### Experimental Pipeline

#### Data Simulation

**Fig. 3:**
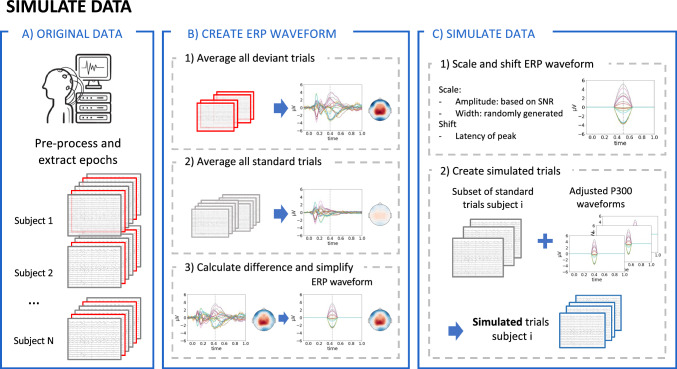
Visual overview of the approach used to generate simulated data. The images used in this overview figure are generated using the P300 dataset, however, the same approach can be used to simulate data based on other datasets and other ERP components. The general approach that is used here is to add an ERP waveform at a known latency to background EEG data. **A** To do this, the original data is first preprocessed and the epochs are extracted for both conditions. **B** The topography of the ERP waveform is then generated by calculating the average response over trials and participants for both conditions separately, after which the difference between both conditions is calculated. The topography at the time of the peak in this difference waveform is then used for the ERP waveform. **C** Finally, the simulated trials are generated by first scaling the amplitude of the ERP waveform according to the SNR and the width. The ERP waveform is then shifted to the correct latency, after which it is added to the data of a standard trial, serving as background EEG data, to construct the deviant trials

In this work, simulated data was needed (1) to train the neural networks and (2) to quantify the performance of the different methods. The general approach that was used is to add an ERP waveform at a known latency to background EEG data. A visual overview of the process described here is shown in Fig. 3. To create the simulated ERP waveform, the average response over trials and participants for both conditions was calculated separately, i.e. for both the standard and deviant stimuli in the oddball paradigm (P300 dataset) and the correct and incorrect stimuli in the SSCT (N400 dataset). The difference between both conditions was then calculated to obtain the topography of the ERP waveform that would be used. The shape of the ERP component was simulated as a half-cycle sinusoidal wave. As the goal was to generate a dataset that resembles the experimental data, the epochs recorded while presenting the standard phoneme (P300 dataset) and the correct sentences (N400 dataset) were used as background EEG data.

Simulated epochs were then generated using the EEG data of all participants in the experimental dataset. For each simulated subject, the EEG data of only one participant was used as background EEG data. To introduce inter-subject variability, the frequency of the sinusoidal waves used for the ERP component was uniformly drawn to obtain a signal length between 100 ms and 300 ms. Furthermore, different uniform latency distributions were simulated for each participant by sampling a mean latency between 350 ms and 550 ms and a standard deviation between 40 ms and 80 ms. For the mean latency, the shape of the grand-average of the experimental data within this time window was used as the sampling distribution, while a uniform distribution was used for the standard deviation. After randomly selecting half of the standard/correct trials, the ERP component was added to the data using latencies sampled from the previously created distribution. To keep the latency of the ERP component within the expected range for healthy controls, trials for which the generated latency was outside the time window of 300 ms to 600 ms were excluded from the dataset. Finally, to increase the amount of generated data, this process was repeated 30 times for each subject.

#### Pipeline for the Performance Evaluation on Simulated Data

The performance of each of the different ERP quantification methods was evaluated on the simulated dataset using different SNRs. The simulated data was based on the P300 experimental dataset, following the method described before. This approach allows generating data with different SNRs by scaling the amplitude of the ERP component that is added to the data. Here, the SNR of the original dataset was determined as the ratio of the power at the peak of the grand-average deviant waveform and the power of the standard trials. Five different SNRs were simulated. For each SNR, the topography of the added ERP component was scaled so that the ratio of the power at the peak of a waveform obtained after averaging the same amount of trials as in the original dataset, and the power of the standard trials was respectively − 6dB smaller, − 3dB smaller, equal, +3dB larger, or +6dB larger compared to the SNR of the original dataset.

As each of the proposed latency estimation techniques needs to train or learn from the data (i.e. defining the template or learning the model parameters), the performance of the different ERP quantification techniques was evaluated using a seven-fold cross-validation approach. The simulated data was split into seven different groups so that all data generated using the original data from a particular subject was included within the same split. This approach guarantees independence between the train and test sets. For each fold, the (initial) template used by the template matching methods was created using only the data in the training set by calculating the difference between the grand averages of the deviant and standard trials between 300 ms and 600 ms and applying a 5 Hz low-pass filter. The obtained template was then used to estimate the latency of the ERP component in the trials of the test set. Similarly, the EEGNet network and the convLSTM network were trained using the data in the training set after which the model was used to estimate the ERP latencies in the test set.

**Fig. 4:**
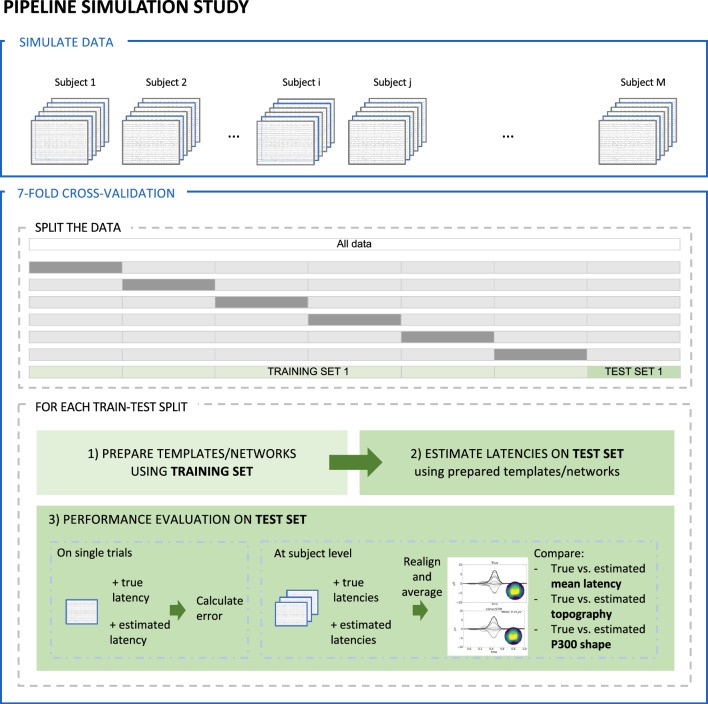
Visual overview of the approach used to evaluate the performance of the different single-trial latency estimation methods using simulated data. In the first step, the simulated data is split into seven different folds. To guarantee the independence between the train and test sets, this was done in a way so that all data generated using the original data from a particular subject was included within the same split. For each fold, the (initial) template are created and the neural networks are trained using only the data in the training set. In the next step, these templates and networks are then used to estimate the latencies of the individual trials in the test set. Finally, the performance of each of the different latency estimation techniques is evaluated. By repeating this process for each of the different folds, the variance across folds can be assessed

Different evaluation criteria were used to evaluate the performance of the methods on the simulated trials. First, to assess the latency estimation in the single trials, the mean absolute error between the true and the estimated latencies was calculated for the different SNRs. This was done by calculating the absolute error for each trial separately and then averaging the errors over all trials and all subjects. The different methods were also evaluated at the level of individual subjects. After estimating the latencies in the single trials, the mean latency was calculated per subject. This approach allows to compare the latencies obtained using the averaging-based methods with those obtained using the single-trial estimation methods. Here, the mean absolute error between the true mean latency and the estimated mean latency was calculated for each subject and each SNR. Also the topography and the shape of the ERP obtained component for the different methods were evaluated. First, the correct topography and shape of the ERP component were calculated by realigning all trials of a specific subject according to the correct latencies. For the averaging-based methods, no realignment was done, and the average waveform was used to quantify the topography and shape of the ERP component. For the single-trial estimation methods, on the other hand, the different trials were realigned according to the estimated latencies before averaging and quantifying the topography and shape of the component. The topographies obtained by the different methods were then compared with the true topography using the mean absolute error. The obtained P300 shapes were compared over the specified time window by calculating the mean absolute error between the true realignment and the realignment based on the estimated latencies. As the SNR of the dataset influences the obtained metrics, the measures were normalized by the absolute amplitude and the area of the true P300 component, respectively, resulting in the relative mean absolute error (RMAE). This approach allows comparing the methods over the different datasets in the simulated data. A visual overview of this pipeline is shown in Fig. 4. The process was repeated for each of the different train-test splits, making it possible to also evaluate if the results are biased by the specific selection of samples within each fold.

Finally, the (realigned) average waveforms of all subjects were realigned to the estimated mean latencies before averaging to obtain the realigned grand-average waveform for each method. Then, the relative absolute error between the true and the estimated grand-average for each of the different methods was calculated to evaluate the obtained shape of the P300 component.

#### Pipeline for the Performance Evaluation on Experimental Data

**Fig. 5:**
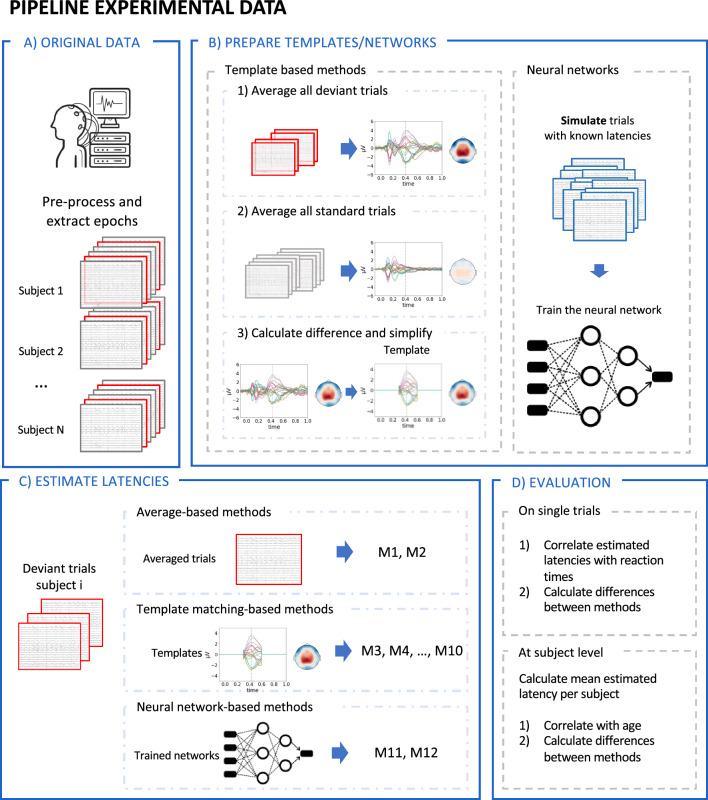
Visual overview of the approach used to estimate the latencies of the individual trials on experimental data. Starting from the pre-processed data, the templates used by the template matching methods are created by calculating the average response over trials and participants for both conditions separately, after which the difference between both conditions is calculated. This difference waveform is then filtered to remove noise and cropped to the time window of interest. For the neural networks, first simulated trials are created following the approach described before, after which the networks are trained using this simulated dataset with known latencies. The obtained templates and trained networks are then used to estimate the latencies of the deviant trials, after which the performance of the different methods is evaluated using different methods depending on the dataset that is used

The different proposed methods for single-trial latency estimation were also applied to both experimental datasets. In these datasets, the true latency of the ERP component in the individual trials is unknown, making it impossible to use this data to train the parameters in the convLSTM network or to use error-based metrics to evaluate the performance of the different methods. Figure 5 gives a visual overview of the pipeline used in this scenario. For the template matching-based methods, the (original) template is created by calculating the difference between averages of the deviant and standard trials across all subjects, after which a 5 Hz low-pass filter is applied to smoothen the template. For the neural network-based approaches, simulated data is created to train the parameters in the networks using the approach described before. The latencies of the ERP component in the experimental trials are then calculated using the trained networks.

**Dataset 1: Oddball Paradigm Eliciting a P300 Component** To evaluate the performance of the methods on the P300 dataset, the approach proposed by Ouyang et al. (2017) was used. Here, the correlation between the estimated latency and the reaction time of the subject was calculated and used to evaluate each of the different methods. This approach is based on the knowledge that, under particular conditions, the neurocognitive processing stream that underlies the stimulus evaluation affects the response time of subjects (Da Pelo et al. 2018). Furthermore, to evaluate the ability of the different methods to estimate the shape of the P300 component on the experimental dataset, all trials were realigned according to the estimated latencies and averaged across all subjects, after which the obtained waveforms were visually compared.

Since the first description of the P300 component in 1965 (Sutton et al. 1965), many researchers have studied the component. An important finding is that the P300 latency is sensitive to neural changes in both development and aging. Different studies have shown that while the P300 latency decreases with age during childhood and adolescence (Polich et al. 1990; Sangal et al. 1998; van Dinteren et al. 2014), it starts increasing again in early adulthood (Rossini et al. 2007; Walhovd et al. 2008; van Dinteren et al. 2014; Brown et al. 1983; Hirayasu et al. 2000). The mean latencies estimated on the experimental data by each of the different methods were used to model the effect of age on the latency of the P300 component and to see which approach results in the best fit of the model. For each method, a linear regression line was fitted to the data and the slope of the curve was then observed to evaluate whether an increase in latency with age was found. The goodness of fit of the regression lines was also evaluated using the root mean squared error (RMSE).

**Dataset 2: SSCT Paradigm Eliciting an N400 Component** As the button press response was delayed in the SSCT task, it is not possible to use the reaction times as a measure to evaluate the single-trial latency estimation methods. Therefore, the effect of age on the latency and the amplitude of the N400 component was investigated using the different single-trial latency estimation methods.

In the paper of Cocquyt et al. (2023), the authors found that the latency of the N400 effect, i.e. the difference between the incorrect and correct evoked responses between 0.3 s and 0.5 s after stimulus onset, was significantly delayed in elderly subjects (ages $\ge$60) compared to both young (ages 20–39) and middle-aged subjects (ages 40–59). Furtermore, also the amplitude of the N400 effect was significantly smaller in elderly subjects compared to young subjects. No significant differences in amplitude were however found between middle-aged and elderly subjects, while this more continuous effect of age on the N400 amplitude was reported by Gunter et al. (1992). In this study, the goal was to replicate these findings and investigate if the changes in amplitude across age groups are caused by changes in the amplitudes of individual trials, changes in the variability in latency, or a combination of both factors. To do this, the latency of the N400 effect was estimated using both the averaging-based methods and the single-trial latency estimation methods. As in this experiment the N400 effect is investigated, the averaged response to the correct trials was subtracted from the incorrect ones for each subject before estimating the latency of the N400 effect. The amplitude of the N400 effect was then calculated as the mean amplitude of the difference in evoked responses between the incorrect and correct conditions within the 0.3 s–0.5 s time window at the Cz electrode for the averaging-based methods. For the single-trial latency estimation techniques, the averaged response to the correct trials was subtracted from the incorrect trials before realignment. The amplitude of the N400 effect was then again calculated as the mean amplitude of this realigned waveform within the 0.3 s–0.5 s time window at the Cz electrode. For each method, the effect of aging on both the latency and the amplitude of the N400 effect was be investigated using a univariate analysis of variance (ANOVA) approach with the age group as an independent variable. Significant main effects were investigated by post hoc multiple comparisons with a Bonferroni correction.

## Results

In this section, the results obtained on the different datasets are discussed. The section starts by giving an overview of the results on the simulated data, where a distinction is made between the performance at the level of the single trials and the performance at the subject level. In the following part, the results obtained on the experimental datasets are discussed. For the oddball task eliciting a P300 component, the performance of the different methods was evaluated by looking at the correlation between the single-trial latencies and the corresponding reaction times of the subjects, by visual inspection of the realigned averaged waveforms, and by investigating the relationship between the mean estimated latency of the component and the age of the subject using regression analysis. For the second experimental dataset, the visual overview of the realigned averaged waveform is again presented, as well as the results of the statistical analyses investigating the effect of age on the latency and the amplitude of the N400 effect.

### Simulated Data

#### Performance at Single-Trial Level

**Fig. 6:**
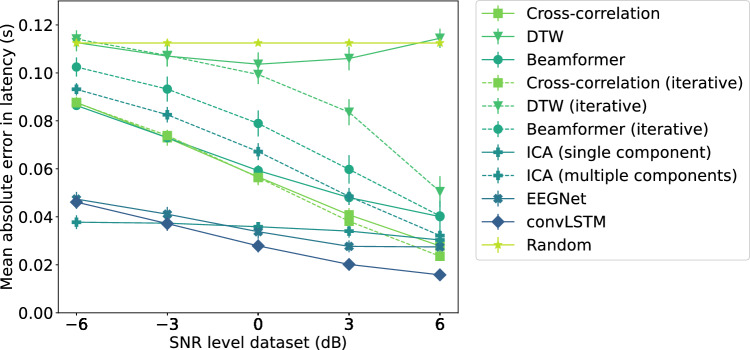
Comparison of the different methods regarding the mean absolute error between the estimated latencies and the true latencies in single trials for each SNR in the simulated data

In Fig. 6, the mean absolute error between the true and the estimated latencies is shown for each single-trial latency estimation method as a function of the SNR level of the trials averaged across the different folds. Also the standard deviation of the mean absolute errors over the different folds is shown. The figure indicates that for the lower SNRs, the neural network-based approaches and single-component ICA outperform all other methods. For higher SNRs, similar performance is also achieved by the cross-correlation based techniques. As expected, in general the estimated latencies improve for higher SNRs. However, only limited improvement is obtained using single-component ICA and even a small decrease in performance is found using DTW-based template matching. Furthermore, the DTW-based approach leads to large errors in the latency estimation for all SNRs, with the method performing only slightly better or even worse than randomly guessing the single-trial latency.

Comparing the template-matching techniques with their iterative variants, the results indicate that the performance of the DTW technique is improved by iteratively updating the template, especially for larger SNRs. Likely, the amplitude difference between the template and the single-trial P300 component becomes smaller, as fewer trials are taken into account when updating the template per subject, improving the performance of the DTW algorithm for the P300 latency estimation. The beamformer, on the other hand, performs worse by updating the template for each subject. Finally, the correlation method and its iterative variant perform similar.

#### Performance at Subject Level

**Fig. 7:**
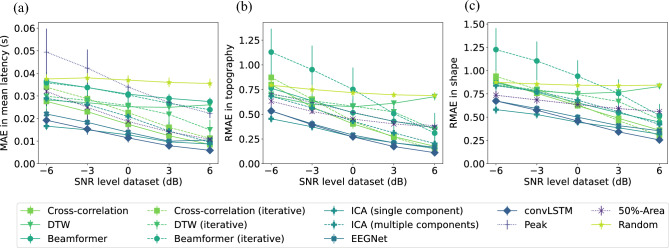
Comparison of the P300 quantification results of the different methods on the simulated datasets on subject level. **A** Mean absolute error between the estimated mean latencies and the true mean latencies for each method and each dataset. **B** Relative absolute error in topography between the estimated topography and the true topography for each method and each dataset. **C** Relative absolute error in shape between the estimated shape of the P300 component and the true shape of the P300 component for each method and each dataset

The different latency estimation approaches were also compared at the level of individual subjects. In Fig. 7A, B and C, respectively, the mean absolute error in mean latency, the relative absolute error in the topography and the relative absolute error between the shapes of the estimated and correct realignments are shown for the different SNR levels of the trials. Looking at the error in the mean latency, the figure shows that the EEGNet network, the convLSTM network and the single-component ICA-based approach all outperform the averaging-based methods. The results also indicate that the peak method results in larger errors in the estimated mean latency and more variability across the different cross-validation folds compared to all other methods, especially for small SNRs.

Figure 7B and C indicates that both the topography and the shape of the estimated P300 component after realignment improve with increasing SNR for all methods except the DTW-based approach. As for the latency estimations, the best results regarding the estimation of topography and shape of the component are obtained using the neural network approaches and the single-component ICA-based method. Furthermore, Fig. 7C indicates that the shape of the P300 component is better approximated using these methods compared to the averaging approach, even for low SNRs. Estimating the latency of the P300 component at the level of single trials thus not only offers more information on the variability of the timing of the P300 component within a subject, but also results in a better estimate of the shape and the topography of the component.

**Fig. 8:**
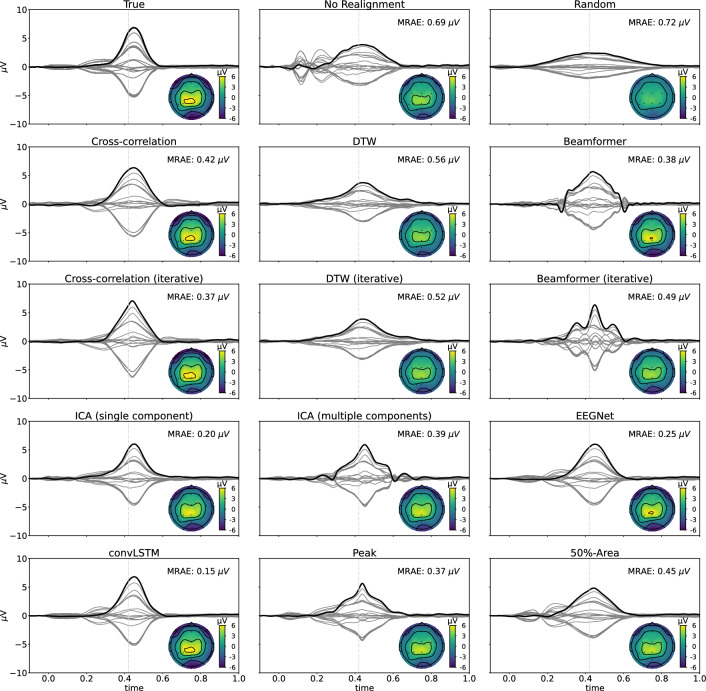
Realignment of the single trials averaged across all subjects with SNR +0dB for each of the different methods. Grey lines represent the different channels, with Pz being marked in black. Also the topography of the realignment at 0.420 s (cf. the dotted vertical grey line) after the stimulus onset is shown. The realigned waveforms are compared to the correct realignment to evaluate how well the shape of the simulated P300 component is estimated. For each method, the mean relative absolute error between the true and the estimated realigned waveforms across all subjects is reported

Finally, in Fig. 8, the realignment of the single trials averaged across all subjects with SNR +0dB is shown for each of the different methods, along with the topography at the time of the peak. Also the non-realigned grand-average and a random realignment are plotted as a reference. The realigned grand-averages are compared with the correct realignment to check how well the shape of the P300 component is estimated by each of the different methods by calculating the mean relative absolute error (MRAE). The figures show that the realignment based on the convLSTM network gives the best results. While the topographies at the peak are very similar across all methods, apart from a scaling factor due to smearing, the shape of the obtained P300 component varies. In the iterative cross-correlation, the (iterative) beamformer and the multiple-component ICA approaches, artefacts are being introduced into the shape of the component due to errors in latency estimations. The figures for the other SNRs are added in appendix C.

### Experimental Data

#### Dataset 1: Oddball Task Eliciting a P300 Component

The performance of each of the different methods on the experimental data was first evaluated by looking at the correlation between single-trial latencies and the corresponding reaction times (Table 3). The highest correlations were found for the neural network approaches, followed by the iterative cross-correlation and the beamformer techniques. Comparing the template matching techniques with their iterative variants, the results indicate that iteratively updating the template improves the correlation value for the cross-correlation method and DTW, which is in line with the results obtained using the simulated data. While the performance of the neural networks and that of the single-component ICA-based approach were similar on the simulated data, the correlation between the latencies estimated by single-component ICA and the reaction times is very low in the experimental dataset. Furthermore, while the performance of single-component ICA was better than the multiple-component approach on the simulated data, the opposite is found for this experimental dataset.

**Fig. 9:**
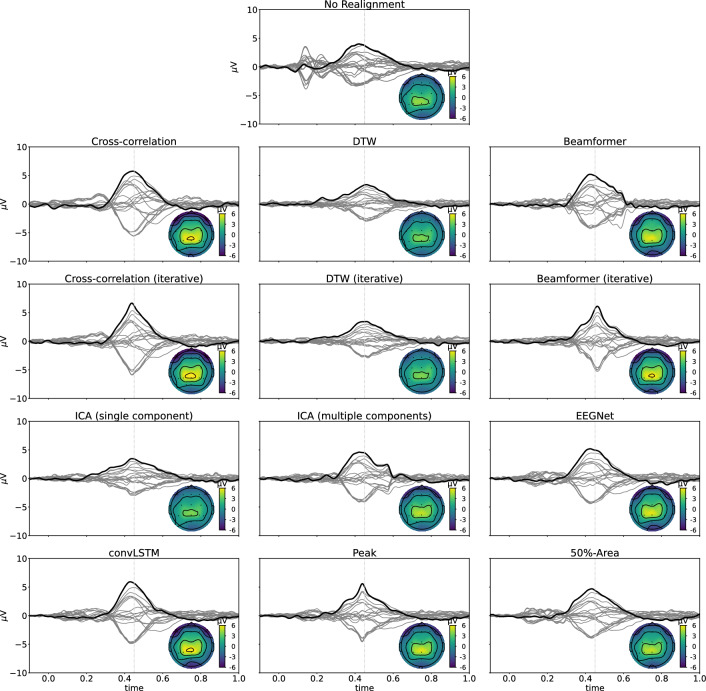
Realignment of the single trials averaged across all subjects in the P300 experimental dataset, obtained with the different methods. Grey lines represent the different channels, with Pz being marked in black. Also the topography of the realignment at 0.420 s (cf. the dotted vertical grey line) after the stimulus onset is shown

Figure 9 shows the realigned averaged waveforms across all subjects, using the estimates of the different methods. Inspection of the figures shows that most single-trial methods result in a more narrow P300 component with a topography similar to that obtained without realignment of the trials. While the realigned waveforms using the averaging-based methods are slightly narrower than the waveform without realignment, the effect is clearer for the single-trial methods, indicating that there is within-subject variability of the P300 latency. The figures also show that the realignments using the DTW- and single-component ICA-based approaches result in a more smeared-out version of the P300 component, resembling the waveform obtained using random latencies in the simulated dataset. This suggests that the latencies obtained using these methods might be incorrect. On the other hand, realigning the epochs using the iterative cross-correlation, the iterative beamformer or the peak methods results in peaks in the realignment, which are similar to the shapes obtained with these methods on the simulated data, indicating that these might be artefacts introduced due to errors in the latency estimation.

**Table 3:** The correlation between the reaction times and the latencies estimated by the different methods for single-trial ERP quantification in the P300 experimental dataset

Method	Correlation with RT
convLSTM	0.30
EEGNet	0.29
Cross-correlation (iterative)	0.26
Beamformer	0.25
Beamformer (iterative)	0.23
Cross-correlation	0.20
ICA (multiple components)	0.17
DTW (iterative)	0.14
ICA (single component)	0.10
DTW	0.08

**Fig. 10:**
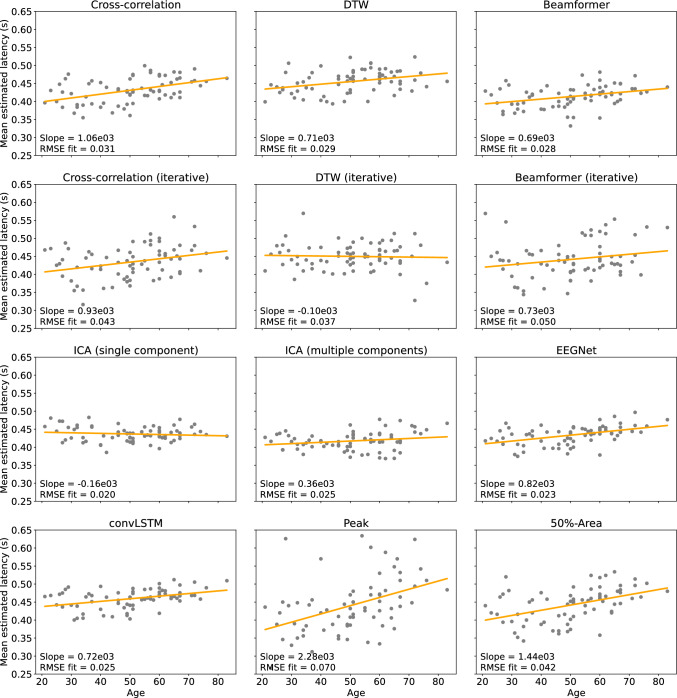
Scatterplot and regression lines of the relationship between the mean estimated latency of the P300 component and the age of the subject for each of the different methods

Finally, the relationship between the mean estimated P300 latency and the age of the subjects for each of the different methods is shown in Fig. 10. For each method, a linear regression line was fitted to the data. The goodness-of-fit of the regression is evaluated using the RMSE, and the slope of the curve is used to evaluate the relationship between the mean estimated P300 latency and the subject’s age. As expected based on literature (van Dinteren et al. 2014), most methods show an increase in estimated P300 latency with age. However, this is not the case for single-component ICA and iterative DTW, where even a decrease is found. The effect is also limited for the multiple-component ICA approach. The strongest increases in latency with age are found for the averaging-based methods and the cross-correlation techniques. Comparing the results of the peak and the 50%-area latency estimation method, a better fit is found for the 50%-area method, as the RMSE is smaller. While these methods result in the largest slope, the best fits to the regression line are found using the neural networks and the ICA-based approaches.

#### Dataset 2: Semantic Sentence Congruity Task Eliciting an N400 Component

**Fig. 11:**
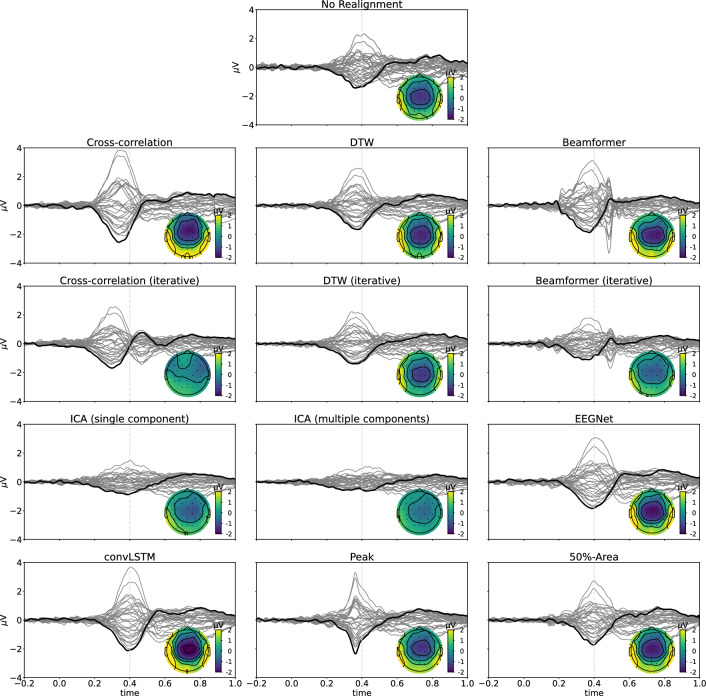
Realignment of the single trials averaged across all subjects in the N400 experimental dataset obtained with the different methods. Grey lines represent the different channels, with Cz being marked in black. Also the topography of the realignment at 0.400 s (cf. the dotted vertical grey line) after the stimulus onset is shown. The realigned waveforms are compared to the correct realignment to check how well the shape of the N400 component is estimated

Also for the SSCT dataset, the realigned averaged waveforms across all subjects are visually compared to evaluate the performance of the different methods. This result is shown in Fig. 11. More narrow ERP components are found when realigning according to the single-trial latency estimates for the neural network-based approaches, as well as for the (iterative) cross-correlation and (iterative) DTW-based template matching methods. The figure also indicates that realignments using the ICA-based techniques result in smeared-out versions of the N400 effect, again resembling the waveform obtained using random latencies in the simulated dataset.

Furthermore, the effect of age on the latency and the amplitude of the N400 effect was investigated. The results obtained using single-trial latency estimation techniques were compared to the traditional averaging approach. To do this, the mean and the standard deviation of the estimated latency were calculated for each subject and the obtained results were compared over the three different age categories: young (ages 20–39), middle-aged (ages 40–59) and elderly (age $\ge$60). The results are shown in Table 4. The original paper by Cocquyt et al. 2023 reported a significant effect of age group on the latency of the N400 effect, with post hoc pairwise comparisons using Bonferroni correction revealing a significant delay in elderly compared to both young and middle-aged subjects. These results are replicated in this study using the 50%-area approach, as was done in the original paper, and similar results are also found using the EEGNet and the convLSTM networks. While significant effects of age are also found using the DTW and iterative beamformer approaches, post-hoc analyses only reported significant delays between elderly and young subjects. Looking at the standard deviation over the estimated latencies per subject, significant effects of age are found using the cross-correlation, beamformer and multiple-component ICA approaches, as well as for the neural networks. Post-hoc analyses reveal increased variations in latency in young subjects compared to middle-aged subjects (EEGNet and convLSTM) as well as compared to elderly subjects (cross-correlation, multiple-component ICA, EEGNet and convLSTM).

Finally, the effect of age on the amplitude of the N400 effect was compared using both the averaging approach and using the estimated latencies to realign the single trials before calculating the amplitude. Here, a significant effect of age was found for the classical averaging approach, as well as after realignment using the (iterative) cross-correlation techniques, the iterative DTW and both neural networks. As in the original paper, post-hoc analyses showed that young subjects had a significantly larger N400 effect compared to elderly subjects (all methods with significant effect of age). However, using the (iterative) cross-correlation methods and the neural networks, also significant differences are found between young and middle-aged subjects, with young subjects having a larger N400 effect.

**Table 4:** Overview of the statistical results on the mean and standard deviations of the estimated latencies, and on the amplitudes of the N400 effect, elicited during the semantic sentence congruity task

	Mean latency		Standard deviation latency		Amplitude	
	F-values	p-values	F-values	p-values	F-values	p-values
Cross-correlation	0.355	ns	7.154	***	4.277	*
DTW	3.828	*	0.167	ns	0.257	ns
Beamformer	2.227	ns	3.284	*	2.528	ns
Cross-correlation (iterative)	1.386	ns	2.024	ns	4.295	*
DTW (iterative)	0.564	ns	1.052	ns	3.831	*
Beamformer (iterative)	3.759	*	1.733	ns	2.060	ns
ICA (single component)	0.872	ns	2.321	ns	1.892	ns
ICA (multiple components)	2.636	ns	4.566	*	1.992	ns
EEGNet	9.355	***	4.581	*	6.585	**
convLSTM	7.308	***	12.360	***	7.230	***
Peak	1.149	ns	NA	NA	5.501	**
50%-area	6.095	**	NA	NA	5.501	**

*p < 0.05, **p <0.01, ***p < 0.001, ns: not significant)

## Discussion

Different methods for the quantification of ERP components were evaluated both on simulated data and on experimental data. The proposed deep learning-based methods, namely the EEGNet approach and the convLSTM network, performed very well on all datasets, proving the applicability of these neural networks to quantify ERP components in single trials. For low SNRs in the simulated dataset, the single-component ICA approach worked slightly better than the neural networks. Nevertheless, only limited differences were found between the estimated latencies, the topographies and the shapes of the ERP components obtained using these approaches.

As expected, in general the estimated latencies improved for higher SNRs in the simulated data. However, a small decrease in performance was found using DTW-based template matching, and only limited improvement was found using single-component ICA. The DTW-based approach led to large errors in the latency estimation for all SNRs, with the method performing only slightly better or even worse than randomly estimating the single-trial latency. This is likely caused by the difference in amplitude of the template and the P300 component in single trials. The template was created by calculating the difference wave between the grand-averages of the deviant and standard trials within a time window and therefore represents a smeared version of the P300 component. As the DTW algorithm calculates the Euclidean distance between the template and the time series to obtain the optimal warping path, this difference in amplitude strongly influences the obtained latency estimates.

For the single-component ICA approach, the only limited improvement for higher SNRs might be caused by the nature of the simulated data, as these simulations were created by adding an independent ERP component with varying amplitude onto background noise. The results suggest that the decomposition algorithm might be able to isolate this ERP component from the data even for very low SNRs, making it probable that a good fit between this component and the template was found. For the multiple-component ICA approach, the performance on the simulated dataset did improve with increasing SNR, but large differences in performance are found compared to the single-component ICA approach. It is likely that by selecting multiple components to create the ERP ICA subspace, also non-ERP-related activity and noise were included, disrupting the time series that were subsequently used to correlate with the template. Furthermore, while single-component ICA gave excellent results on the simulated dataset for all SNRs, the performance of this method is much lower on the experimental data, where the data is more complex. There is more variability in the topography of the ERP component, resulting in a mismatch between the template and the selected ICA component containing the ERP. This is further confirmed by the differences in performance between the single- and multiple-component ICA approaches, as the multiple-component ICA approach performed better than the single-component approach on the experimental data.

The performance of the single-trial latency estimation techniques was also compared to that of averaging-based approaches in terms of the estimated latencies, and the topography and shape of the obtained ERP component after realignment. The results on both the experimental datasets and the simulated data showed that the neural network-based approaches typically performed better than the averaging approaches. Single-trial latency estimation approaches thus do not only offer more information on the variability of the timing of the component, but also result in better estimates of the shape and topography of ERP components. The results also clearly indicated that when using averaging-based approaches, the 50%-area based approach should be preferred over the peak-based method. The drawback of this averaging approach, however, is still that it is unable to capture the correct shape of the ERP component and that it does not provide information on the variability of the latency of the ERP component.

The added value and the usability of single-trial latency estimation using the neural networks was further proven on the SSCT dataset, where a larger N400 effect was found in young subjects compared to both middle-aged and elderly subjects, while only significant differences between young and elderly subjects were found using the averaging approach in both the original study by Cocquyt et al. Cocquyt et al. (2023) and this work. This amplitude reduction of the N400 effect with age from middle-age on, was previously shown by Gunter et al. (1992), and Cocquyt et al. (2023) attributed the discrepancy in the results to differences in the age range under investigation. However, the current results using the neural networks show that the effect of age on the amplitude of the N400 effect is indeed present in the data from the middle-age on. Furthermore, by including the information from the standard deviation of the estimated latencies in the single trials, which was found to be larger in younger subjects compared to both the middle-aged and elderly subjects, the results were able to confirm that the significant changes in the amplitude of the N400 effect are indeed due to changes in amplitude and are not caused by latency jitter of the N400 effect. This is also in line with the findings of Hoffman and Morcom (2018), who reported reduced activity of in some regions of the typical left-hemisphere semantic network which have been reported as potential generators of the N400 effect, namely the inferior prefrontal, posterior temporal and inferior parietal cortex, in older subjects compared to the younger. These findings show the added value of including single-trial latency estimations in the analysis of the data.

In this work, different neural networks were adapted to quantify the ERP component in single trials and compared to other methods commonly used in literature using both simulated and experimental data. Even though the simulated dataset was created as realistic as possible, certain assumptions, such as the topography and simplified shape of the ERP component, influence the obtained dataset and the performance of the different methods. Furthermore, these assumptions also affect the results obtained on the experimental dataset. As no information on the component latency is present in the experimental data, the parameters of the neural networks can only be learned based on simulated data. This is an important limitation of deep learning approaches for single-trial ERP component quantification. If the assumptions about the shape or the topography of the ERP component in the simulated data are incorrect, the network will not be able to perform well on the experimental data. The need for the simulated data also limits the applicability of the networks on datasets where the ERP components of interest are not known a-priori. In this case, data-driven approaches, such as topographic analyses of variance and microstate analyses, have a clear advantage, as they allow estimating the ERPs without being limited to one peak selected beforehand. Another remark that can be made is that the same ERP component may have different characteristics in different populations. In this case, however, it would be possible to train different networks for the different populations, or to include characteristics of the different populations under investigation in the simulated data, thereby creating a more robust neural network. Also more advanced methods to generate the simulated data could be used, for example using ICA to extract one or more subcomponents of the ERP component from the original data and using them as ERP waveform that is added to the background EEG data that is used to train the network. A final limitation of the neural networks is that they work as a so-called black box, returning an estimate of the latency of the ERP component without giving insight into what the network’s decision is based upon. This makes the deep learning approaches less interpretable compared to other methods, such as the (iterative) cross-correlation method. Lastly, it is important to note that each method always returns an estimate of the component latency even when no ERP component is present in the data. Therefore, it could be useful in future work to combine the ERP component classifiers used in BCIs with these latency estimation techniques.

## Conclusion

Two deep learning approaches were proposed for single-trial latency estimation of ERP components. Application of these methods on both simulated and experimental data has shown that the neural networks outperform other single-trial latency estimation methods, suggesting that deep learning techniques can be used as a new approach to estimate the latency of ERP components in single trials. More specifically, the proposed methods for quantifying the ERP components resulted in better estimates of the topography and the shape of the components. On the P300 experimental data, higher correlations were found between the P300 single-trial latencies and the subjects’ reaction times. Furthermore, using the N400 dataset, a larger N400 effect was obtained in the young subjects compared to both the middle-aged and elderly subjects, while significant differences were only found between the young and elderly subjects using the averaging approach. By including the information from the standard deviation of the estimated latencies in the single trials, the results showed that the significant changes in the amplitude of the N400 effect are indeed due to changes in amplitude and are not caused by latency jitter of the N400 effect, proving the added value of the neural networks for single-trial latency estimation compared to the averaging-based approaches. While the EEGNet network and the convLSTM network are more complex than other techniques proposed in literature, they allows researchers to better study the trial-to-trial latency variability of the ERP component, even in data with a low SNR. A drawback, however, is that simulated data needs to be created upfront to train the network, limiting the applicability of the proposed network to study ERP components for which information is limited. In future work, the proposed neural network approach could be applied both to other ERP components, as well as to the data of other populations where the ERP components may have different characteristics, to further study its validity.

## Figures and Tables

**Fig. 12:**
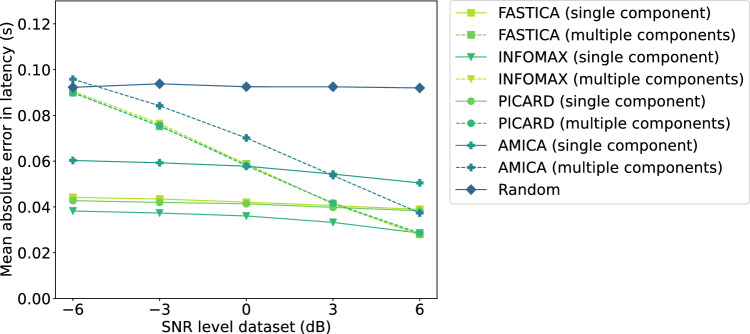
Comparison of the different ICA decomposition algorithms (extended Infomax, Picard, fastica and AMICA) regarding the mean absolute error between the estimated latencies and the true latencies in single trials for each each SNR in the simulated data

**Fig. 13:**
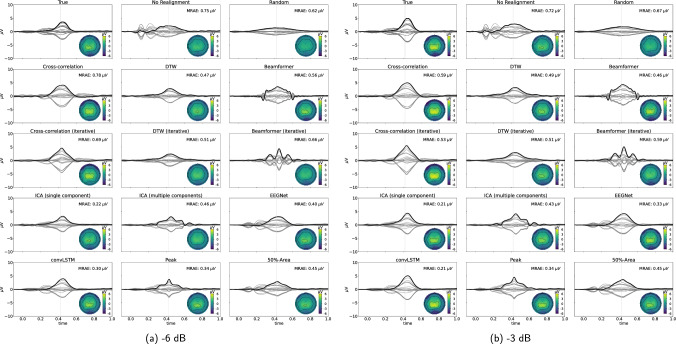
Realignment of the single trials averaged across all subjects with SNR -6dB and -3dB for each of the different methods. Grey lines represent the different channels, with Pz being marked in black. Also the topography of the realignment at 0.420 s (cf. the dotted vertical grey line) after the stimulus onset is shown. The realigned waveforms are compared to the correct realignment to evaluate how well the shape of the simulated P300 component is estimated. For each method, the mean relative absolute error between the true and the estimated realigned waveforms across all subjects is reported

**Fig. 14:**
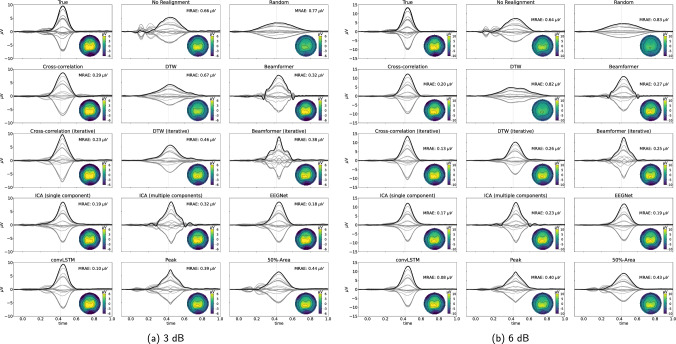
Realignment of the single trials averaged across all subjects with SNR +3dB and +6dB for each of the different methods. Grey lines represent the different channels, with Pz being marked in black. Also the topography of the realignment at 0.420 s (cf. the dotted vertical grey line) after the stimulus onset is shown. The realigned waveforms are compared to the correct realignment to evaluate how well the shape of the simulated P300 component is estimated. For each method, the mean relative absolute error between the true and the estimated realigned waveforms across all subjects is reported

## Data Availability

The datasets presented in this article are not readily available because of privacy issues. The code for the latency estimation models can however be provided upon request. Requests to access the code should be directed to emma.depuydt@ugent.be.

## A Methods for ERP Component Quantification

### A.1 Averaged Trial ERP Component Quantification

Two different techniques were used to quantify the ERP component’s latency after averaging, namely the peak latency and the 50%-area latency.

**M1: Peak Latency** The most commonly used technique for measuring the latency of ERP components is by defining a time window and finding the latency of the maximal value in this time window at a specific electrode. For the P300 component, the signal at the Pz electrode between 250 and 650 ms post-stimulus was used, while for the N400 component the focus was on the Cz electrode using the 200 to 600 ms time window. These measurement windows were selected based on visual inspection of the data averaged across subjects (Luck 2014), while the Pz and Cz electrodes were chosen as the P300 and N400 components typically reach maximum values over the parietal and central electrodes, respectively (Polich 2012).

**M2: 50%-Area Latency** An alternative method for calculating the component latency on the averaged waveform is by calculating the area under the ERP waveform over a specified time window and then finding the time point where a fraction of this area is reached (Luck 2014). In this work, the 50%-area was used. Similar to the calculation of the peak latency, the time window from 250 to 650 ms post-stimulus was considered for the P300, and 200 ms to 600 ms for the N400 components. The ERP waveform were again evaluated at the Pz and Cz electrodes for the P300 and N400 components, respectively.

### A.2 Single Trial ERP Component Quantification Based on Template Matching

Seven different approaches were selected for the single-trial latency estimation based on template matching: non-iterative and iterative template matching using cross-correlation, non-iterative and iterative template matching using DTW, a non-iterative and an iterative spatiotemporal LCMV beamformer and template matching after ICA decomposition using both a single component and multiple components.

**M3: Template Matching Using the Cross-Correlation Curve** In this method, the resemblance between a template and the single trials was measured by calculating the correlation between both at each time lag. This corresponds to calculating the cross-correlation curve between the template and the signal. To take into account the spatial information present in the data, the approach proposed by Ouyang et al. (2017) was used. Here, cross-correlation curves were calculated for all electrodes, after which they were averaged. The optimal latency was then determined as the latency corresponding to the peak in this averaged cross-correlation curve.

**M4: Template Matching Using Subsequence Dynamic Time Warping (DTW)** A second template-matching approach that was used is based on DTW. Different variants of the DTW algorithm exist that differ in the posed constraints. In the original version, one of these constraints is the boundary condition, which states that the first and the last indices of the first sequence must be matched with the first and last indices of the second time series in the alignment (Müller 2007). However, the aim in this work is to find a subsequence, i.e. a template, within a longer sequence, namely the EEG signal. Therefore, the subsequence DTW variant, in which this constraint is dropped, was used as implemented in the tslearn library (Tavenard et al. 2020). The optimal alignment between the template and the single trial is expressed as a mapping between the time indices of the two signals. The latency of the ERP component was estimated as the time point to which the peak latency of the template is matched in the optimal alignment path.

**M5: Spatiotemporal LCMV Beamformer** The spatiotemporal linearly constrained minimum variance (LCMV) beamformer is a flexible spatiotemporal filter developed by van Vliet et al. (2016) to estimate the amplitude of ERP components. In this work, their method was extended to allow estimation of the latency of the ERP component by shifting the template in time. For each time-shift, the amplitude of the ERP component was estimated after which the time-shift with the highest amplitude was selected as the latency of the ERP component in the single trial.

**M6, M7 and M8: Iterative Approaches** The single-trial latency estimation algorithms M3, M4 and M5 can be extended by iteratively updating the template. This approach was first described for the cross-correlation method by Woody in 1967 (Woody 1967) and allows the estimation of a subject-specific template. In each iteration, the different trials were realigned based on the estimated latencies to obtain a subject-specific estimate of the ERP component. However, incorrectly estimated latencies can influence and distort the shape of the estimated component. Therefore, a weighted average of the old template (80%) and the subject-specific component estimate (20%) was used as the new template in the subsequent iteration.

**M9 and M10: Template Matching After Independent Component Analysis** Multiple ICA decomposition approaches can be used for the latency quantification of the ERP component in single trials. The first choice to be made is which specific ICA algorithm to use for the decomposition. Algorithms typically used for EEG data include FastICA (Hyvarinen 1999), extended Infomax (Lee et al. 1999), Picard (Ablin et al. 2018) and adaptive mixture ICA (AMICA) (Palmer et al. 2012; Delorme et al. 2012). Secondly, the ERP component can either be considered as a single peak within the ICA decomposition, or as a combination of several components, each with an independent topography, which are mixed at the scalp level due to volume conduction (Onton et al. 2006). In the first case, the latency of the ERP component can be determined by selecting the ICA component with the highest cross-correlation with the template and choosing the corresponding time lag as the estimated latency. In the second case, several components from the ICA decomposition are combined before calculating the cross-correlation with the template to determine the latency of the ERP component. To determine which components to take into account, i.e. which subspace of the ICA components form the ERP component, two different criteria were used: the correlation between the IC topography after backprojection to the scalp and the template should be positive, and the p-value should be less than 0.01 (Ouyang et al. 2017).

The different ICA algorithms and the two latency quantification approaches were evaluated using the simulated data. These results can be found in appendix B. Based on these results, the remainder of this work focusses on the extended Infomax ICA algorithm using both the single- and multiple-component based approaches.

## B Template Matching Using ICA: Comparison of Algorithms and Methods

Multiple ICA decomposition approaches for the latency quantification of the ERP component in single trials were compared in this work using the simulated data. The ICA algorithms that were considered in this work included FastICA (Hyvarinen 1999), extended infomax (Lee et al. 1999), Picard (Ablin et al. 2018) and adaptive mixture ICA (AMICA) (Palmer et al. 2012; Delorme et al. 2012). Furthermore, also the effect of using only a single ICA component for the latency estimation or using a combination of multiple components was compared. In Fig. 12, the mean absolute error between the true and the estimated latencies are shown in function of the SNR level of the dataset. The figure shows that when only a single ICA component is considered, the SNR level of the dataset has a limited influence on the performance of the latency estimation. This might be caused by the nature of the simulated data, as a single independent P300 component was added to background noise. In this case, the extended Infomax algorithm performs best, and only small differences are found between the fastICA and Picard implementations. A clear trend is however found between the performance of the methods and the SNR of the data when considering multiple ICA components. Here, the extended Infomax, fastICA and Picard implementations gave almost identical results for all SNRs, with the performance of the AMICA algorithm being slightly worse. This trend is very similar to the one observed for the cross-correlation latency estimation method (Fig. 6). Therefore, it is probable that by selecting multiple ICA components, not only the ERP component but also noise is included in the combined signal, which leads to larger errors in the single-trial latency estimation for lower SNRs. Based on these results, the decision was made to use the extended Infomax algorithm for the further comparison of the different latency estimation methods.

## C Realigned Grand-Averages of the Simulated Data for Different SNRs

In Figs. 13 and 14 the realignment of the single trials averaged across all subjects for each of the different SNRs are shown, along with the topography at the time of the peak, comparing the different latency estimation techniques. Also the non-realigned grand-average and a random realignment are plotted as a reference. The realigned grand-averages are compared with the correct realignment to check how well the shape of the P300 component is estimated by each of the different methods by calculating the mean relative absolute error (MRAE). The figures show that the realignment based on the convLSTM network gives the best results. Similar results are found across the different SNRs. While the topographies at the peak are very similar across all methods, apart from a scaling factor due to smearing, the shape of the obtained P300 component clearly varies. In the iterative cross-correlation, the (iterative) beamformer and the multiple-component ICA based approaches, artefacts are being introduced into the shape of the ERP component due to errors in latency estimations.
